# Effects of intracellular calcium accumulation on proteins encoded by the major genes underlying amyotrophic lateral sclerosis

**DOI:** 10.1038/s41598-021-04267-8

**Published:** 2022-01-10

**Authors:** Giovanni De Marco, Annarosa Lomartire, Umberto Manera, Antonio Canosa, Maurizio Grassano, Federico Casale, Giuseppe Fuda, Paolina Salamone, Maria Teresa Rinaudo, Sebastiano Colombatto, Cristina Moglia, Adriano Chiò, Andrea Calvo

**Affiliations:** 1 Department of Neuroscience, ALS Centre, “Rita Levi Montalcini”, University of Turin, Via Cherasco 15, 10126 Turin, Italy; 2 Neurology Unit 1, Azienda Ospedaliero-Universitaria Città della Salute e della Scienza di Torino, Via Cherasco 15, 10126 Turin, Italy; 3 Department of Oncology, University of Turin, via Michelangelo 27/b, 10126 Turin, Italy; 4 Neuroscience Institute of Turin (NIT), Via Verdi, 8, 10124 Turin, Italy; 5 Institute of Cognitive Sciences and Technologies, C.N.R., Via S. Martino della Battaglia, 44, 00185 Rome, Italy

**Keywords:** Biochemistry, Proteases, Proteolysis, Immunoblotting, Neurological disorders, Autophagy, Mechanisms of disease, Proteolysis

## Abstract

The aetiology of Amyotrophic Lateral Sclerosis (ALS) is still poorly understood. The discovery of genetic forms of ALS pointed out the mechanisms underlying this pathology, but also showed how complex these mechanisms are. Excitotoxicity is strongly suspected to play a role in ALS pathogenesis. Excitotoxicity is defined as neuron damage due to excessive intake of calcium ions (Ca^2+^) by the cell. This study aims to find a relationship between the proteins coded by the most relevant genes associated with ALS and intracellular Ca^2+^ accumulation. In detail, the profile of eight proteins (TDP-43, C9orf72, p62/sequestosome-1, matrin-3, VCP, FUS, SOD1 and profilin-1), was analysed in three different cell types induced to raise their cytoplasmic amount of Ca^2+^. Intracellular Ca^2+^ accumulation causes a decrease in the levels of TDP-43, C9orf72, matrin3, VCP, FUS, SOD1 and profilin-1 and an increase in those of p62/sequestosome-1. These events are associated with the proteolytic action of two proteases, calpains and caspases, as well as with the activation of autophagy. Interestingly, Ca^2+^ appears to both favour and hinder autophagy. Understanding how and why calpain-mediated proteolysis and autophagy, which are physiological processes, become pathological may elucidate the mechanisms responsible for ALS and help discover new therapeutic targets.

## Introduction

Amyotrophic lateral sclerosis (ALS) is a progressive and fatal neurological disease that primarily affects upper and lower motor neurons. Currently, there are neither reliable biomarkers nor effective pharmacological treatments for the disease and its pathogenesis is still poorly understood. The discovery of genetic aetiology in ≈70% of familial and ≈10% of non-hereditary ALS cases pointed out possible causes of motor neurons degeneration. However, ALS cases linked to genetic mutations are less than 10–15% and these mutations affect more than 30 genes encoding for proteins that have disparate functions^1,2^. In fact, some of these proteins act by binding DNA and/or RNA [TAR DNA binding protein-43 (TDP-43), fused in sarcoma/translocated in liposarcoma (FUS), matrin-3^3–5^]; some are enzymes [Cu/Zn superoxide dismutase 1 (SOD1), and valosin-containing protein (VCP)^6,7^]; others are implicated in protein degradation (p62/sequestosome-1)^8^, contribute to the formation of cytoskeleton (profilin-1)^9^ or regulate intracellular trafficking pathways [chromosome 9 open reading frame 72 (C9orf72)]^10^.

A lot of evidence supports the hypothesis that excitotoxicity is one of the toxic conditions at the heart of motor neuron degeneration in ALS^11–13^. The excitotoxic process is defined as neural cell damage caused by an abnormal intake of calcium ions (Ca^2+^) due to the hyperactivation of ionotropic glutamate receptors^14^. This Ca^2+^ overload may contribute to necrotic or apoptotic cell death through mitochondrial dysfunctions, aberrant production of reactive oxygen species and/or endoplasmic reticulum stress^15–17^.

The aim of the current study is to analyse the effects of intracellular Ca^2+^ accumulation on proteins codified by some of the most important genes responsible for ALS, in order to find possible metabolic processes common to all or most of these proteins. For this purpose, the protein profile of TDP-43, FUS, matrin-3, SOD1, VCP, p62/sequestosome-1, profilin-1 and C9orf72, will be evaluated in three different cell types – SK-N-BE(2), HeLa and peripheral blood mononuclear cells (PBMC) – induced to accumulate Ca^2+^ in their cytoplasm. Accumulation will be reached by inducing an excessive ion intake or by altering ion flux between intracellular storage structures and cytoplasm.

## Results

### Calpain-mediated cleavage of proteins codified by genes related to ALS

Firstly, we investigated whether the proteins linked to ALS considered in this study are substrate of calpains, a class of proteins the activity of which is strictly dependent on Ca^2+^. For this purpose, whole lysates of SK-N-BE(2) cells were treated with exogenous active recombinant human calpains-1 and -2 and the effects of the two proteases on endogenous proteins were evaluated.

**Figure 1:**
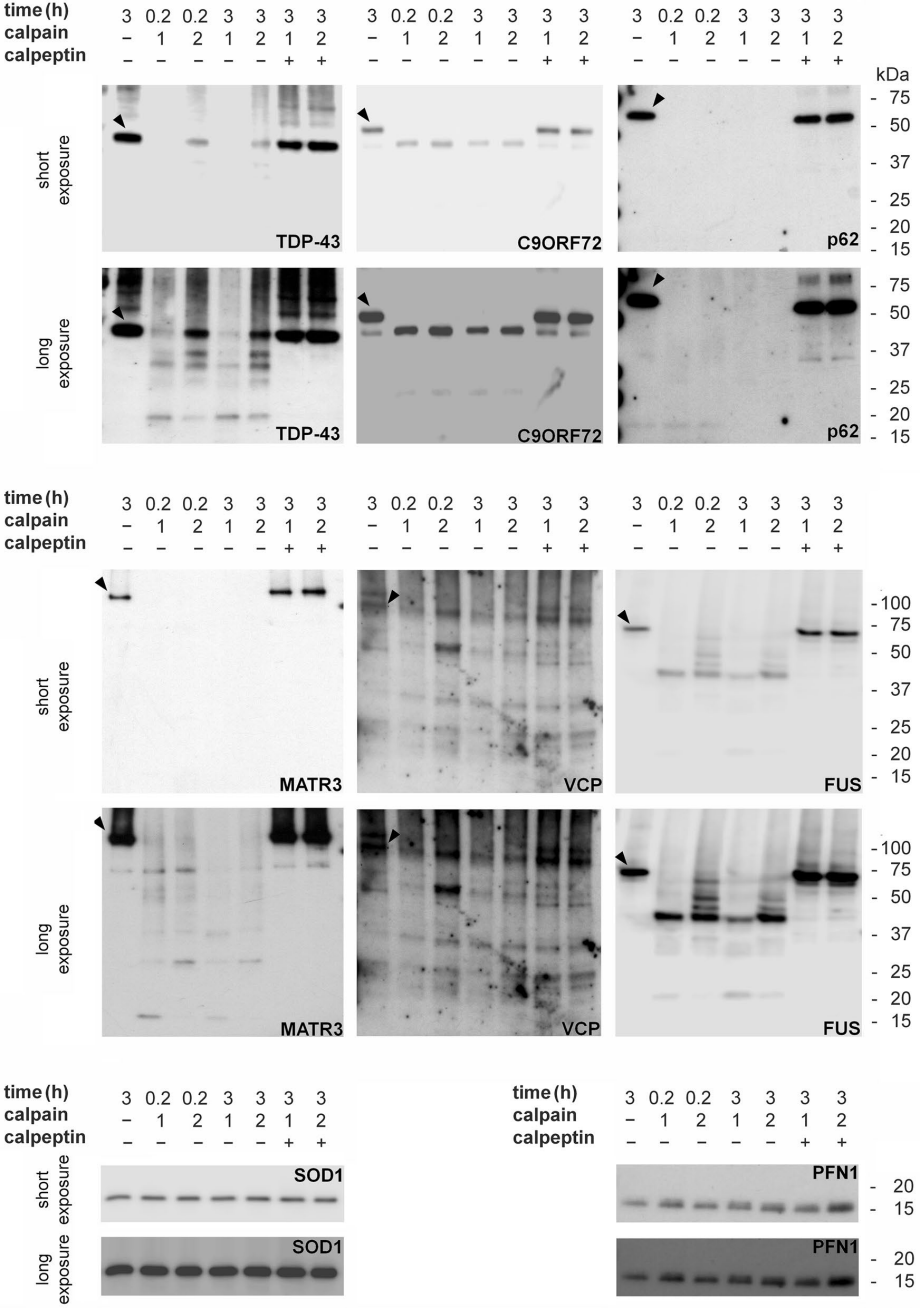
Calpain cleavage of proteins coded by genes linked to ALS. Western immunoblot analysis of TDP-43, C9orf72 (C9ORF72) p62/sequestosome-1 (p62), matrin-3 (MATR3), VCP, FUS, SOD1 and profilin-1 (PFN1) evaluated, as endogenous protein, in SK-N-BE(2) cell lysate incubated with 2 U of recombinant active human calpains-1 or -2, at 37 °C for 10 or 180 min. Incubation of the cell lysate in presence of 20 mM calpeptin, a calpain inhibitor, was also performed. Blots are representative of three independent experiments. The arrowhead indicates the immunoreactive band corresponding to the full-length protein.

TDP-43 (45 kDa), C9orf72 (50 kDa), p62/sequestosome-1 (65 kDa), matrin-3 (117 kDa), VCP (97 kDa) and FUS (75 kDa) are substrates of calpains, because the levels of the respective full-length protein decreased already at 10 min of treatment with the proteases (Fig. 1, Table 1).

**Table 1:** Calpains and caspases involved in the proteolysis of proteins codified by genes linked to ALS.

	Calpain-1*	Calpain-2*	Caspase-3	Caspase-6	Caspase-7	Caspase-8
TDP-43	++++	+++	+++	++++	++++	+++
C9orf72	++++	++++	+	++	++	+++
p62/sequestosome-1	++++	++++	++	++++	++	++++
Matrin-3	++++	++++	++	++++	++++	++++
VCP	+++	+	−	++	−	++
FUS	++++	++++	++	+++	+++	+++
SOD1	−	−	−	−	−	−
Profilin-1	−	−	−	−	−	−

*At 10 min of treatment.

− ,  no decrease in full-length protein or decrease ≤ 5%;  + ,  5% < decrease in full-length protein ≤ 10% or decrease in full-length protein < 5% with visible cleavage products;  ++ ,  10% < decrease in full-length protein ≤ 50%;  +++ ,  50% < decrease in full-length protein ≤ 90%;  ++++ ,  decrease in full-length protein > 90%.

Proteolytic products were revealed by antibodies against TDP-43, C9orf72, matrin-3, VCP and FUS (Fig. 1). In detail, cleavage of TDP-43 generated at least four fragments with a molecular mass of about 36, 32, 25 and 18 kDa, in agreement with a previous study^18^, reporting that three of these fragments are the calpain-mediated proteolytic products with amino acid sequences 1–324, 1–286 and 1–243. Cleavage of C9orf72 generates two proteolytic products at ≈45 and 25 kDa. Matrin-3 antibody identified some fragments, with molecular mass of ≈70, 25 and 15 kDa. Interestingly, treatment with calpain-2 at 10 min determined a modest decrease in full-length VCP amount, but gave rise to an evident proteolytic band at ≈50 kDa (Fig. 1, Table 1). This proteolytic fragment is probably better recognised than the full-length protein by the antibody used. Calpain-generated fragments of FUS ranged from few kDa less than the full-length protein to ≈20 kDa. The most evident of these fragments had a molecular mass of ≈45 kDa (Fig. 1). Antibodies against SOD1 (18 kDa) and profilin-1 (15 kDa) detected no decrease of protein amount as well as no generation of proteolytic fragments (Fig. 1, Table 1).

### Caspase-mediated cleavage of proteins codified by genes related to ALS

Differently from calpains, caspases are not strictly dependent on Ca^2+^ for their activity, but Ca^2+^ is one of the stimuli that trigger apoptosis, a form of programmed cell death that ends in the activation of these proteases. Thus, we verified whether the proteins linked to ALS considered in this study are substrate for caspases-3, -6, -7 and -8, which are the most important apoptotic caspases. Here too, whole lysates of SK-N-BE(2) cells were treated with exogenous active recombinant human caspases above mentioned and the effects on endogenous proteins were evaluated.

**Figure 2:**
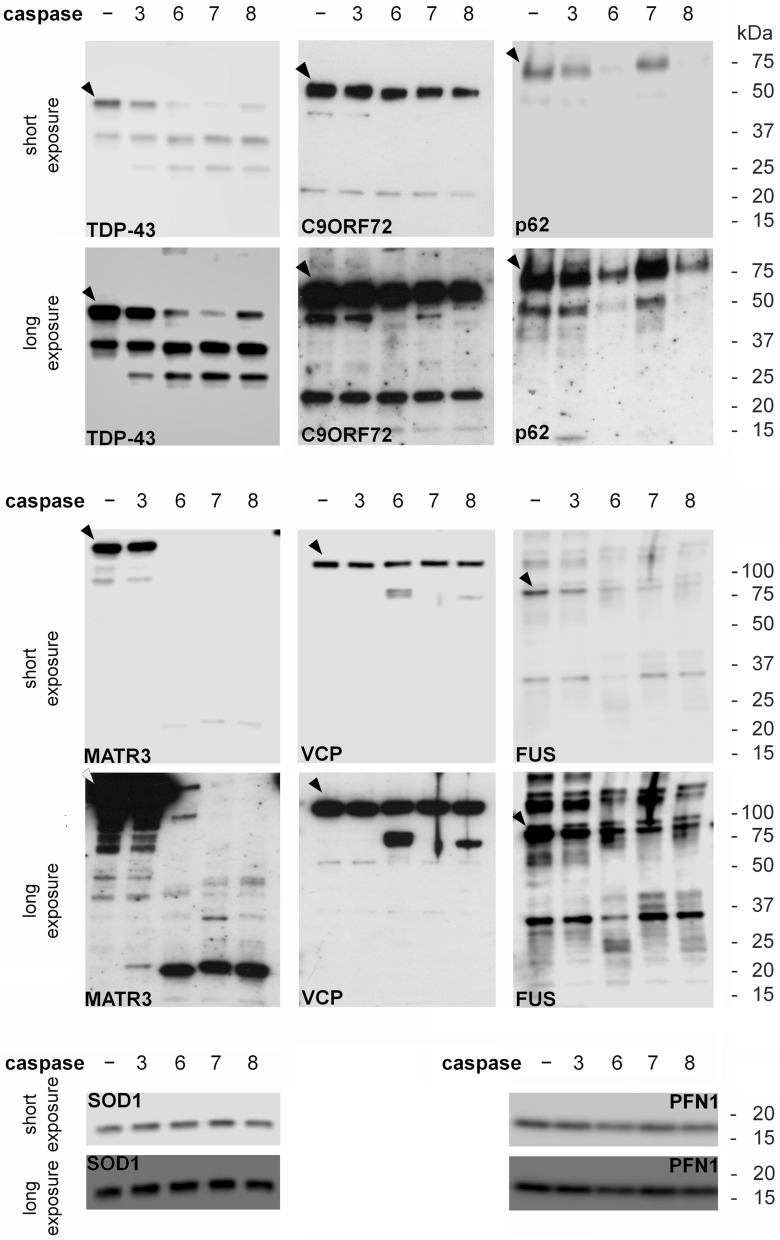
Caspase cleavage of proteins coded by genes linked to ALS. Western immunoblot analysis of TDP-43, C9orf72 (C9ORF72) p62/sequestosome-1 (p62), matrin-3 (MATR3), VCP, FUS, SOD1 and profilin-1 (PFN1) evaluated, as endogenous protein, in SK-N-BE(2) cell lysate incubated with 2 U of recombinant active human caspases-3, 6, 7 or -8, for 3 h at 37 °C. Blots are representative of three independent experiments. The arrowhead indicates the immunoreactive band corresponding to the full-length protein.

A decrease in the amount of full-length TDP-43, C9orf72, matrin-3 and FUS was clearly detected when the cell lysate was treated with caspase-3, -6, -7 or -8 (Fig. 2, Table 1). Incubation of SK-N-BE(2) lysate with caspases-6 or -8 determined a more evident decrease in full-length p62/sequestosome-1 levels respect to treatments with caspases -3 and -7 (Fig. 2, Table 1), in line with the results of a previous study^19^. The most important fragments generated by caspase cleavage of TDP-43 had a molecular mass of 35 and 25 kDa. The first one corresponds to the amino acid sequence 90–414 at the C-terminus of the protein; the second one should be the 220–414 proteolytic product described by Zhang et al^20^ or the 170–414 fragment reported by us and others^21,22^. The loss in the amount of full-length matrin-3 was associated with the formation of a fragment at about 20 kDa (Fig. 2). This product is consistent with the 681–847 C-terminal fragment generated by caspase cleavage at the consensus site DETD^680^, previously described^23^. Incubation of cell lysate with caspases-6 and -8 caused a slight decrease in the full-length VCP but determined the generation of a proteolytic fragment at about 50 kDa (Fig. 2, Table 1). A previous paper^24^ showed that the cleavage of VCP by the two above mentioned caspases occurs at the consensus site VAPD^179^. Finally, SOD1 and profilin-1 appear not to be substrates for the caspases here used, since neither decrease in the amounts nor proteolytic products of the proteins were detected (Fig. 2, Table 1).

### Profile of proteins codified by ALS-related genes in SK-N-BE(2) treated with ionomycin or thapsigargin

The profile of the proteins linked to ALS was subsequently evaluated in SK-N-BE(2) cells induced to raise their cytoplasmic Ca^2+^ levels. The proteins were analysed in cultured cells incubated with two concentrations (1 µM or 5 µM) of ionomycin (a Ca^2+^ ionophore) or thapsigargin (a sarcoplasmic/endoplasmic reticulum Ca^2+^-ATPase inhibitor).

**Figure 3:**
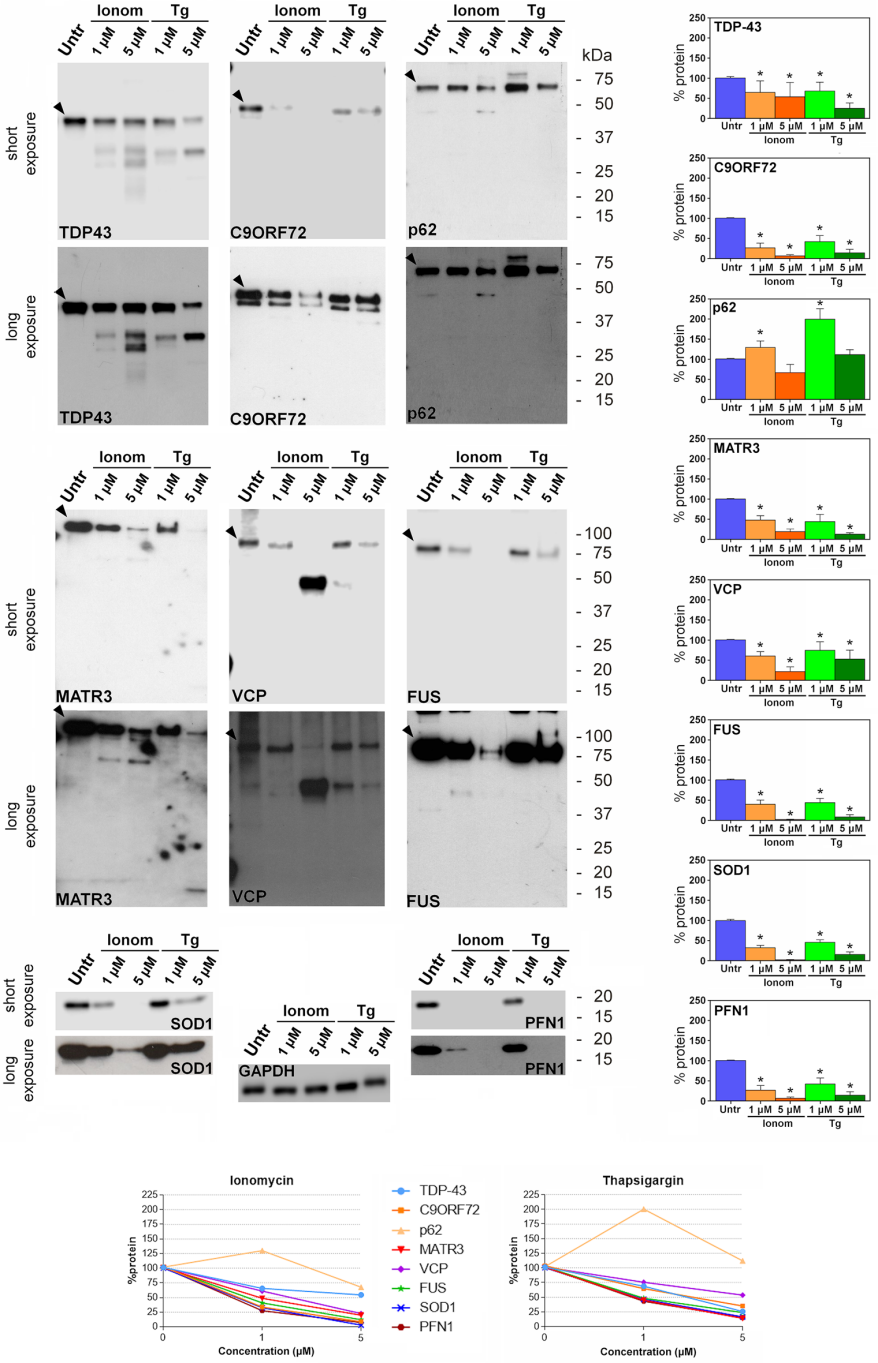
Effects of increased cytoplasmic Ca^2+^ in SK-N-BE(2) cell line. Western immunoblot analysis of TDP-43, C9orf72 (C9ORF72) p62/sequestosome-1 (p62), matrin-3 (MATR3), VCP, FUS, SOD1 and profilin-1 (PFN1) in SK-N-BE(2) cell line incubated with ionomycin (Ionom) or thapsigargin (Tg) at two different concentrations (1 mM and 5 mM) for 24 h. GAPDH expression is used as a measure of equal protein loading. The arrowhead indicates the immunoreactive band corresponding to the full-length protein. Blots are representative of three independent experiments. The bar graphs illustrate the expression of each protein treated with ionomycin or thapsigargin compared to the expression in untreated cells (Untr), with the latter being arbitrarily considered as 100%. The line graphs show the trend comparison among the levels of the proteins in cells treated with ionomycin or thapsigargin. Data are representative of three independent experiments. *p < 0.05; error bars = standard deviations.

Treatment of cells with ionomycin determined a significant reduction in the amount of TDP-43, C9orf72, matrin-3, VCP, FUS, SOD1 and profilin-1, which is larger in cells exposed to 5 µM ionomycin. Instead, treatment of SK-N-BE(2) cells with 1 µM ionomycin caused an increase of 30% in p62/sequestosome-1 levels compared to untreated cells, while incubation with 5 µM ionomycin resulted in a decrease of ≈30% in the protein amount, although this percentage is not significant (Fig. 3).

Ionomycin treatment of SK-N-BE(2) gave rise to 36, 32, 25 and 18 kDa species of TDP-43, to 70 kDa fragment of matrin-3, to 70 kDa product of VCP (5 µM of ionomycin) and to 45 kDa fragment (barely visible) of FUS. These species overlap the proteolytic products observed in cell lysate following treatment with calpains (Figs. 1, 3).

Treatment of SK-N-BE(2) with thapsigargin led to a significant decrease in the amount of TDP-43, C9orf72, matrin-3, VCP, FUS, SOD1 and profilin-1 (Fig. 3, Table 1). The higher the concentration of thapsigargin, the larger the decrease in the levels of these proteins was. Instead, treatment with 1 µM thapsigargin doubled the level of p62/sequestosome-1. In cells exposed to 5 µM thapsigargin, the amount of p62/sequestosome-1 was similar to that observed in untreated cells (Fig. 3). The proteolytic products detected by the antibodies against TDP-43 (35 and 25 kDa) and matrin-3 (20 kDa, with 5 µM of thapsigargin) overlap the proteolytic species observed in cell lysates following treatment with recombinant caspases (Figs. 1, 3). In cell incubated with 1 µM thapsigargin TDP-43 (32 kDa) and VCP (50 kDa) fragments generated by calpains were slightly visible (Fig. 3).

### Profile of proteins codified by ALS-related genes in HeLa and PBMC

The profile of the proteins previously evaluated in SK-N-BE(2) cells was then studied in other two cell types, HeLa and PBMC, using the same treatments applied in SK-N-BE(2) cells.

**Figure 4:**
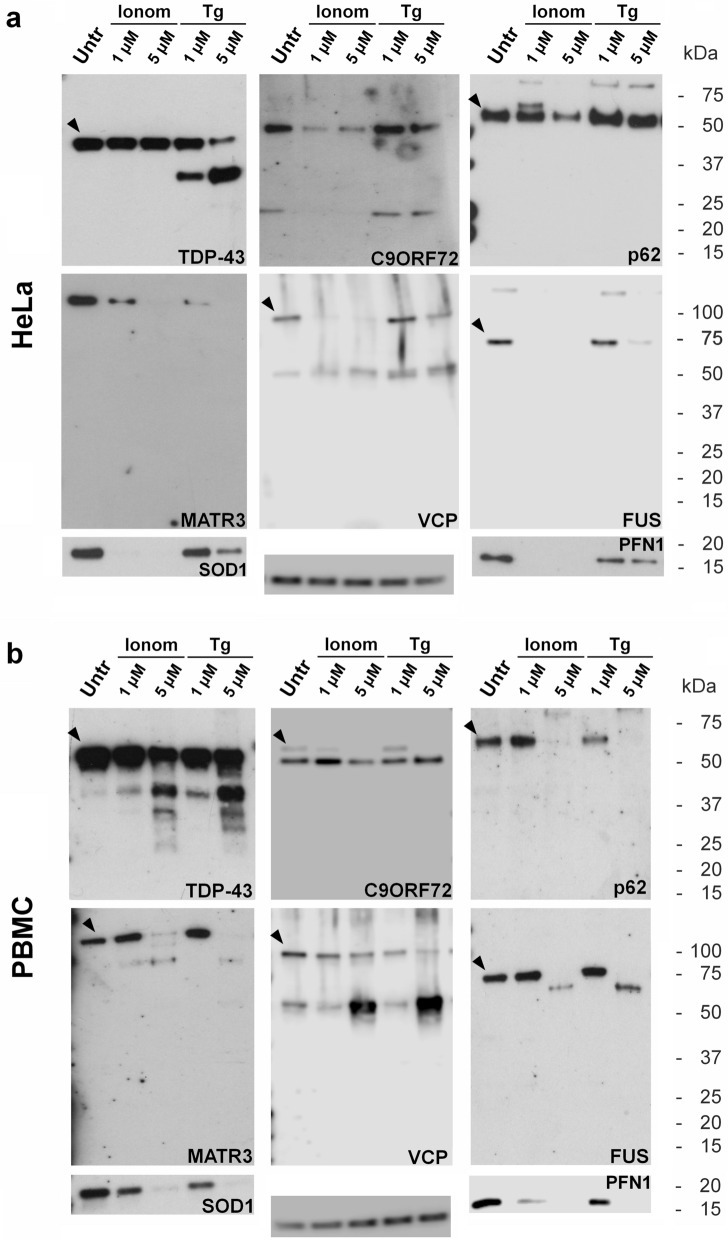
Effects of increased cytoplasmic Ca^2+^ in HeLa cell line and in peripheral blood mononuclear cells (PBMC). Western immunoblot analysis of TDP-43, C9orf72 (C9ORF72) p62/sequestosome-1 (p62), matrin-3 (MATR3), VCP, FUS, SOD1 and profilin-1 (PFN1) in HeLa **(a)** as well as PBMC **(b)** incubated with ionomycin (Ionom) or thapsigargin (Tg) at two different concentrations (1 mM and 5 mM) for 24 h. GAPDH expression is used as a measure of equal protein loading. The arrowhead indicates the immunoreactive band corresponding to the full-length protein. Blots are representative of three independent experiments. Untr:  untreated cells.

The results obtained in HeLa cells overlapped the ones obtained in SK-N-BE(2), with the exception that ionomycin treatment did not induce the formation of proteolytic products generated by calpains (Fig. 4a).

In PBMC, immunoreactive band corresponding to the full-length C9orf72 was less appreciable than the 45 kDa proteolytic band, also in untreated cells. Incubation of PBMC with both ionomycin and thapsigargin gave rise to the formation of calpain-generated proteolytic products in the proteins that are substrates of calpains. Caspase-generated proteolytic products were detected for none of proteins here analysed (Fig. 4b).

Of note, the 45-kDa band obtained when FUS is cleaved by calpains might be present in PBMC of a patient carrying FUS c.1509_1510delAG mutation (Supplementary Fig. S1).

### Profile of other proteins not directly linked to ALS

The following proteins are not coded by genes linked to ALS, but an evaluation of their profile is useful to identify pathways triggered by intracellular Ca^2+^ accumulation.

**Figure 5:**
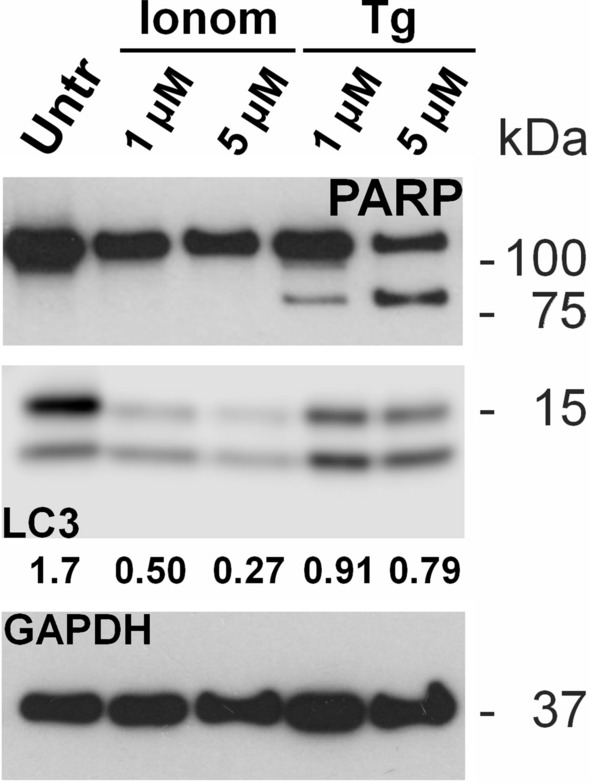
Effects of increased cytoplasmic Ca^2+^ on proteins not directly linked to ALS. Western immunoblot analysis of a protein linked to apoptosis, PARP-1 (PARP), and a protein linked to autophagy (LC3) in SK-N-BE(2) cell line treated with ionomycin (Ionom) or thapsigargin (Tg) at two different concentrations (1 mM and 5 mM) at 37 °C for 24 h. The ratio between LC3-I (non-lipidated form) and LC-II (lipidated form) is reported. GAPDH expression is used as a measure of equal protein loading. Blots are representative of three independent experiments. Untr : untreated cells.

Since thapsigargin incubations appeared to induce proteolysis by apoptotic caspases, the activation of the latter was confirmed by evaluating the profile of PARP-1. In fact, when PARP-1 is cleaved by caspases, a proteolytic C-terminal product of 89 kDa is generated^25^. Here, this fragment was observed in SK-N-BE(2) cells, when exposed to thapsigargin at 1 µM and was even more evident, together with a drastic loss of the full-length protein, when treated with 5 µM thapsigargin (Fig. 5).

The opposite trend in the expression of p62/sequestosome-1 respect to the other proteins here analysed raised the question of a modulation of autophagy by the treatments here applied. Conversion of LC3 protein from the non-lipidated (LC3-I) to the lipidated (LC3-II) form, with the latter being detectable at a lower mass than the former, is widely used to monitor autophagy^26^. In untreated SK-N-BE(2) cells, the LC3-I/LC3-II ratio was > 1, whereas it was < 1 when treated with ionomycin or thapsigargin. The higher the concentration of these compounds, the lower the LC3-I/LC3-II ratio. Furthermore, treatment with ionomycin or thapsigargin was associated with a decrease in the total amount (LC3-I + LC3-II) of LC3 (Fig. 5).

### Time-dependent treatments

For a better understanding of the pathways triggered by intracellular Ca^2+^ overload and the consequent effects on the proteins linked to ALS, the profile of some of the proteins was analysed in SK-N-BE(2) cells exposed for 2, 8 and 24 h to 1 μM ionomycin or thapsigargin. The decrease in the amount of TDP-43 and profilin-1 (and PARP-1) induced by ionomycin and thapsigargin was already appreciable after 2 h of treatment. Calpain-mediated fragments of TDP-43 were detected already at 2 h of ionomycin exposure. The 35 kDa fragment of TDP-43 and the 89 kDa product of PARP-1 generated by caspases were appreciable after 24 h of thapsigargin treatment.

**Figure 6:**
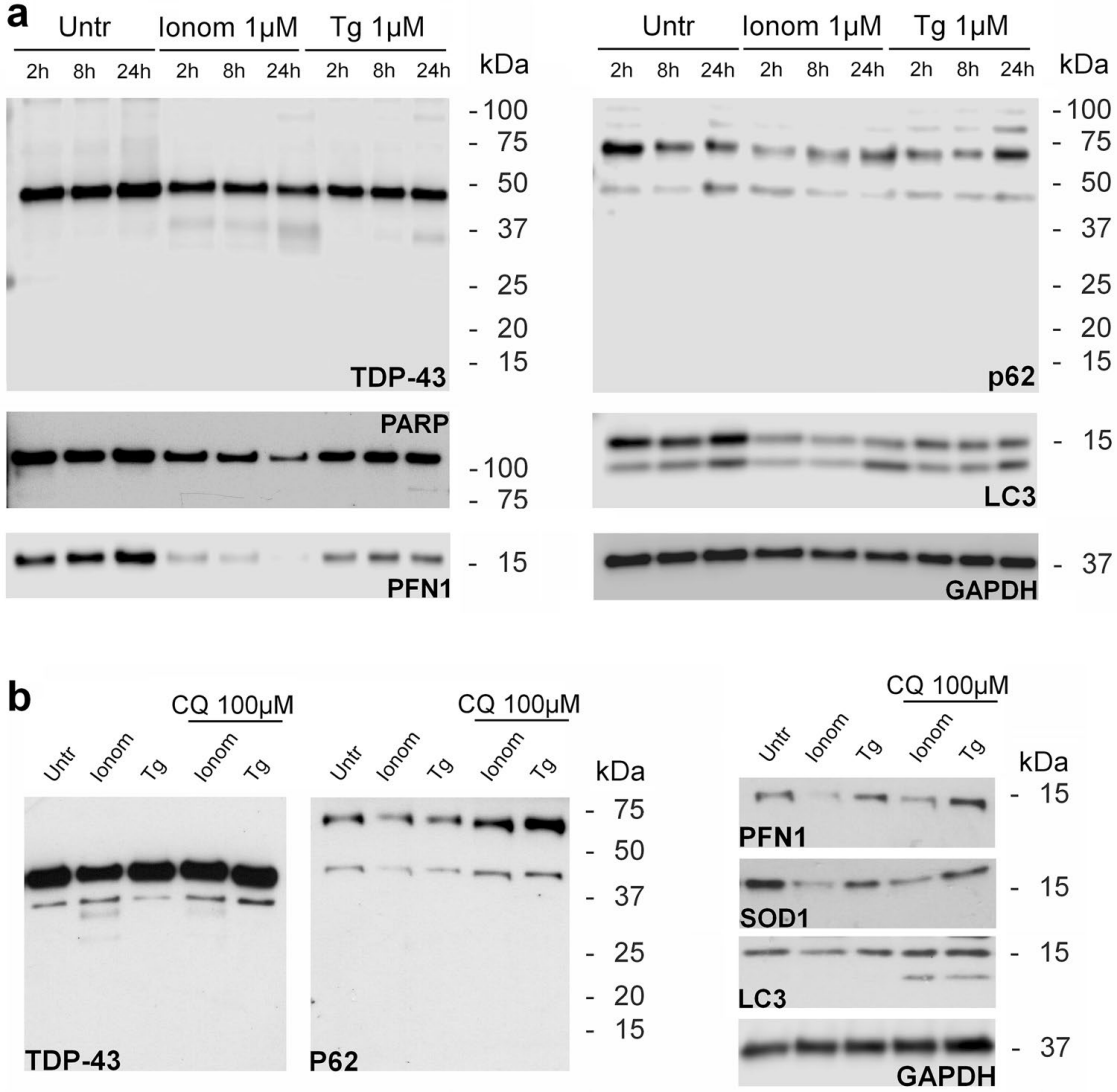
Effects of intracellular Ca^2+^ accumulation over time or in presence of chloroquine in SK-N-BE(2) cells. Western immunoblot analysis of TDP-43, p62/sequestosome-1, profilin-1 (PFN1), PARP-1 (PARP) and LC3 in SK-N-BE(2) cell line treated with 1 mM ionomycin (Ionom) or thapsigargin (Tg) for 2, 8 and 24 h **(a)**. Western immunoblot analysis of TDP-43, p62/SQSTM1, profilin-1, SOD1, PARP-1 and LC3 in SK-N-BE(2) cell line treated with 1 mM ionomycin (Ionom) or thapsigargin (Tg) for 2 h with and without preincubation with 100 μM chloroquine (CQ) **(b)**. GAPDH expression is used as a measure of equal protein loading. Blots are representative of three independent experiments. Untr:  untreated cells.

In untreated cells, p62/sequestosome-1 levels decreased during the time interval considered (2–24 h), and the LC3-I/LC3-II ratio was > 1 without appreciable variations. Treatment with ionomycin or thapsigargin caused an even more drastic decrease in p62/sequestosome-1 and LC3 levels at 2 h compared to untreated cells. At the same time, the LC3-I/LC3-II ratio decreased respect to the ratio calculated in untreated cells. However, both treatments raised the overall p62/sequestosome-1 amount in the time interval considered (2–24 h). In this period, the LC3-I/LC3-II ratio decreased and became about 1 or less in cells treated for 24 h (Fig. 6a).

### Preincubation with chloroquine

In order to confirm the involvement of autophagy in the decrease of the amount of the proteins codified by genes linked to ALS, preincubation of SK-N-BE(2) with chloroquine, an inhibitor of the autophagic process, was performed for 2-h treatments with ionomycin or thapsigargin.

These treatments prevent, at least partially, the loss of TDP-43, SOD1 and profilin-1. In both ionomycin- and thapsigargin-treated cells, preincubation with chloroquine resulted in an increase in p62/sequestosome-1 levels as well as favoured the formation of the lipidated form of LC3 respect to untreated cells (Fig. 6b).

Figure S2 reports the experiments performed and the main results obtained.

## Discussion

The discovery of genes associated with ALS is in constant progress and undoubtedly important for the comprehension of the causes underlying the disease. However, the numerous functions of the proteins coded by these genes show how complex the mechanisms involved in the pathology are. This study describes the effects of intracellular Ca^2+^ overload, one of the processes strongly suspected to play a role in motor neuron degeneration, on proteins coded by the most relevant genes associated with ALS, in the attempt to find pathways that are common to these proteins.

The investigation here reported discloses that elevated intracellular Ca^2+^ concentrations result in a decrease in the levels of the proteins examined except for p62/sequestosome-1. Calpain- and caspase-mediated proteolysis as well as autophagy are the processes involved in the regulation of the amount of these proteins. The predominance of one of these processes appears to depend on the cell type. Indeed, calpain activity was poorly appreciable in HeLa, whereas caspase activity was not found in PBMC. This cell type-specificity makes it difficult to establish which of the processes here identified play a role in degeneration of motor neuron. However, increasing evidence supports a role for calpains- and caspases- mediated proteolysis as well as of autophagy in ALS onset and progression.

Calpains belong to a class of proteases whose catalytic activity is strictly dependent on Ca^2+^^27^. Here, cytoplasmic Ca^2+^ accumulation caused by a massive ion influx or, to a lesser extent, by internal storage impairments, was seen to activate calpains. TDP-43, C9orf72, p62/sequestosome-1, matrin-3, VCP and FUS are substrates for calpains. Calpain-1 plays a protective role in the early phase of ALS but its prolonged activity may be harmful for motor neurons^28^. Furthermore, a selective inhibitor of calpains has been demonstrated to be neuroprotective in a mouse model of ALS^29^.

Caspases are a class of proteases essential for apoptosis, a form of programmed cell death^30^. Differently from calpains, caspases are not strictly dependent on Ca^2+^ for their activity, but Ca^2+^ is one of the stimuli that trigger various mechanisms leading to the activation of these proteases. Our study found that cytoplasmic Ca^2+^ accumulation, when caused by internal storage alterations, activates caspases. This activation occurs later in time with respect to that of calpains when mediated by Ca^2+^ influx. TDP-43, matrin-3, FUS and, to a smaller extent, C9orf72 are good substrates for caspases. p62/sequestosome-1 is a better substrate for caspases-6 and -8 than for -3 and -7. VCP is cleaved only by caspases-6 and -8. The lack of the proteolytic fragment of VCP generated by caspases-6 and -8 suggests that intracellular Ca^2+^ accumulation activates caspases-3 and -7 only. A contribution of caspases in the pathogenesis of ALS has been well documented^31,32^. However, caspase-6 appears to play a neuroprotective role^33^.

The amount of SOD1 and profilin-1 dramatically decreased in cells induced to accumulate Ca^2+^ in their cytoplasm, although these proteins were found to be not substrates for calpains and caspases. The increase of p62/sequestosome-1 suggested the hypothesis of an involvement of autophagy as a consequence of intracellular Ca^2+^ overload. Autophagy is a degradation/recycling process that plays a wide variety of roles in the cell, including regulation of protein turnover, elimination of unwanted components, defence towards invading microorganisms, and provision of nutrient elements^34^. The link between Ca^2+^ and autophagy is well documented but controversial. In fact, a rise in intracellular Ca^2+^ levels can enhance but also inhibit the autophagic flux^35–37^. p62/sequestosome-1 is a cargo protein that binds to proteins targeted for degradation through autophagy^8,38^. When autophagy occurs, p62/sequestosome-1 is itself degraded, together with the proteins it carries^8^. Instead, when autophagic process is blocked, the levels of p62/sequestosome-1 may rise because of the block of its degradation. The block of autophagic flux is confirmed when accumulation of p62/sequestosome-1 is paralleled by accumulation of lipidated LC3^39^. Our results show that high amounts of intracellular Ca^2+^ lead initially to a decrease in p62/sequestosome-1 amount, which is followed by an accumulation of the protein. This accumulation is accompanied by the conversion of non-lipidated LC3 to the lipidated form. These findings indicate that intracellular Ca^2+^ accumulation initially enhances autophagy, but later blocks the process. When autophagy is active, all the proteins linked to ALS here considered, including SOD1 and profilin-1, are degraded. In fact, preincubation with an autophagic inhibitor prevent, at least partially, the loss of these proteins. However, the block of autophagy in the later stages is not associated with a recovery of the degraded proteins, with the exception of p62/sequestosome-1 (the levels of which appear to be modulated by Ca^2+^ through autophagy rather than proteolysis by calpains and caspases). A possible explanation is that, in the persistence of intracellular Ca^2+^ accumulation, the cell attempts to maintain the autophagic activity (even if the process is blocked), thus continuing to produce the necessary proteins. At the same time, the synthesis of the proteins degraded by autophagy is arrested. Autophagy is an important factor in the pathogenesis of ALS, but its role is extremely complex. In fact, this process undoubtedly helps in eliminating intracellular misfolded proteins and protein aggregates, which are pathological features of ALS-affected neurons. However, an inadequate but also an excessive autophagic flux have been linked to ALS pathology, and autophagy may either exacerbate or alleviate the disease processes at different stages^40–42^.

Some of the effects induced by intracellular Ca^2+^ accumulation on the proteins associated with ALS here studied are described in motor neurons, animal or cell models of ALS. Calpain- and caspase-generated species of TDP-43 are a feature of motor neurons in ALS-affected subjects^18,43^. These proteolytic fragments, because of their high propensity to aggregate, are a determinant for motor neuron toxicity^44,45^. Accumulation of p62/sequestosome-1 takes place in spinal cord of a mouse model of ALS^46^ and has also been associated with TDP-43 cleavage and aggregation^47^. Decrease in the levels of the protein is one of the hypotheses linking the repeat hexanucleotide expansion in the non-coding region of *C9orf72* to neurodegeneration^48,49^. Our study suggests that this pathological loss of C9orf72 can also be determined by intracellular accumulation of Ca^2+^. Loss of neuronal VCP has been associated with neurodegeneration and TDP-43 pathology in VCP KO mice^50^.

The presence of mutations on the genes coding the proteins here investigated in ALS-affected patients opens the question of whether the effects induced by intracellular Ca^2+^ overload are different on mutant respect to wild-type proteins. Mutations in TDP-43 may favour its cleavage by both calpains and caspases^18,51^. Mutant VCP and SOD1, besides p62/sequestosome-1 may interfere with autophagic process^52^. Furthermore, our study found that one of the calpain-generated proteolytic fragments of FUS appears to be present in PBMC of a patient carrying a frameshift mutation on the codifying gene, suggesting that mutations in FUS may make the protein more prone to calpain cleavage. Thus, the effects of calpain- and caspase-dependent cleavage as well as autophagy may be modified by the presence of mutations in the proteins here analysed.

**Figure 7:**
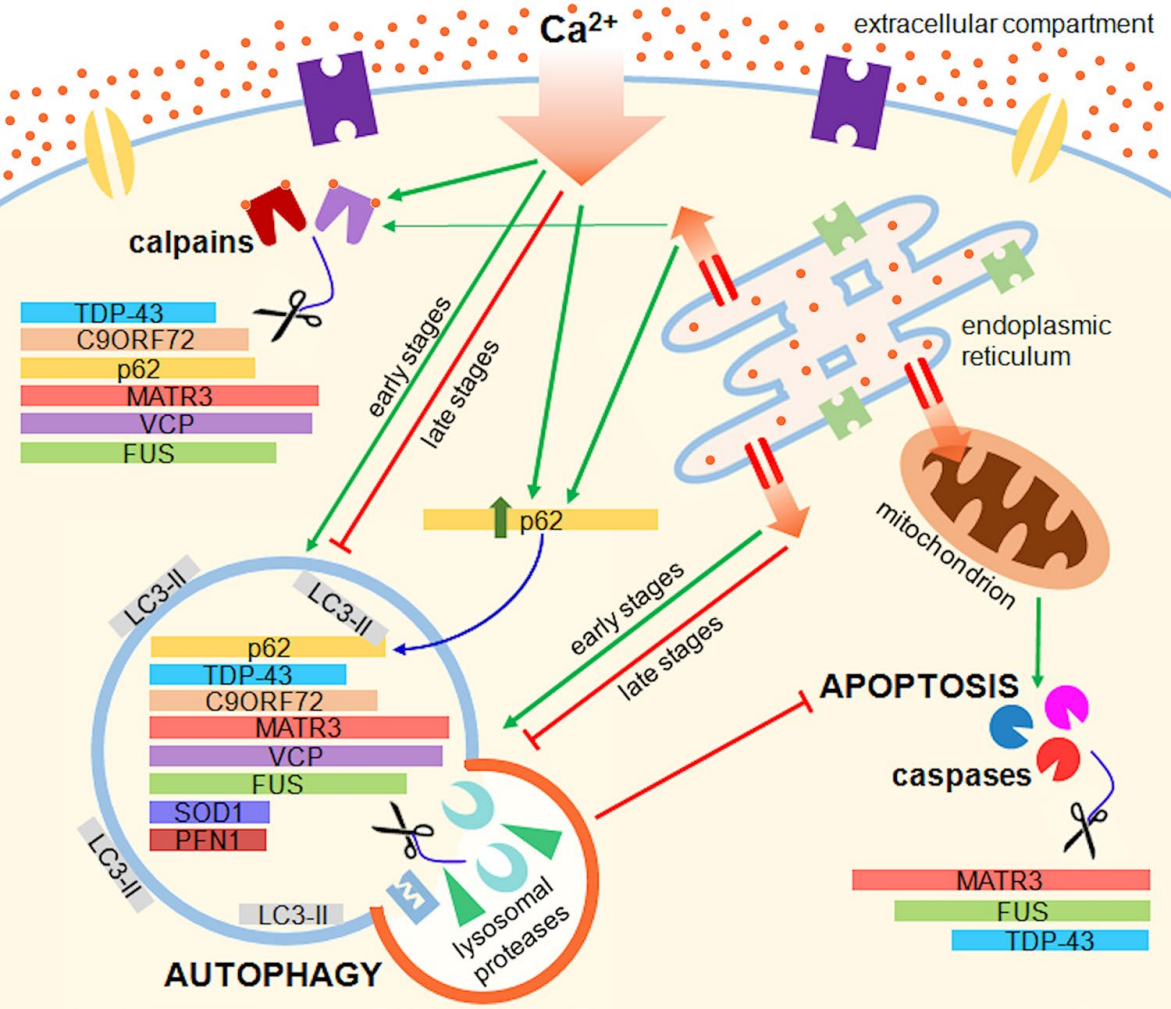
Effects of intracellular Ca^2+^ accumulations on proteins linked to ALS. Excessive influx as well as abnormal release from intracellular storages (i.e., endoplasmic reticulum) of Ca^2+^ causes the activation of proteolytic processes including calpain and caspase cleavage as well as autophagy. In the early stages, the raise in intracellular Ca^2+^ levels triggers the activation of calpains and favours autophagy. Over a longer period, intracellular Ca^2+^ accumulation leads to a block of the autophagic process, which causes an accumulation of p62/sequestosome-1 and results in the activation of apoptotic caspases. Apoptosis can also be triggered by Ca^2+^ accumulation in mitochondria.

However, the decrease in the levels of the protein here studied not always mirrors what is known to happen in motor neurons of ALS-affected patients. In fact, FUS^53^ and profilin-1^54^ induce neurodegeneration through a gain- rather than a loss-of-function property. Acquisition of toxic properties by SOD1 appears to be more probably involved in ALS pathogenesis than loss of antioxidant activity, although the latter may play a modifying role in the disease^55^. Matrin-3 may induce neurodegeneration with either loss or gain of function^56^. In motor neurons of subjects affected by ALS, loss of TDP-43 occurs in the nucleus and is often accompanied by cytoplasmic accumulation^57^ and not by an overall decrease of the protein amount in the cell. Likely, this study describes only a small part of a complex machinery set in motion by Ca^2+^. We have to consider that Ca^2+^, as a second messenger, controls many other metabolic pathways that may be affected by the perturbation of its homeostasis^58^. The complexity of this homeostasis is also exemplified by the cleavage of TDP-43 by caspases that occurs when Ca^2+^ concentration is not only high but also low^21^. Furthermore, as previously reported, raised intracellular Ca^2+^ levels may have opposite effects on autophagic flux. Moreover, some of the proteins linked to ALS here analysed (i.e. VCP and C9orf72, besides p62/sequestosome-1) play themselves an important role in the control of the processes that determine their degradation^59,60^. Finally, calpain-mediated proteolysis, apoptosis and autophagy are tightly connected. In fact, calpains can both regulate the autophagic flux^35,61^ and may activate or inactivate caspases^62,63^, as well as a block of autophagy leads to apoptosis and thus to caspase activation^64,65^ (Fig. 7).

What emerges from this study is that accumulation of Ca^2+^ in the cell, which is likely to be at the core of motor neuron degeneration in ALS, causes the alteration of a complex and delicate balance that leads to the activation of proteolytic processes. These processes may target the proteins coded by genes linked to the pathology (Fig. 7). If we exclude apoptosis, that is a form of cell death, calpain proteolysis and autophagy are physiological processes, which may also have protective functions for the cell. Further and deeper investigation is required to link the findings of this study with the mechanisms underlying ALS pathogenesis. In particular, understanding how and why the mechanisms here identified become pathological as well as evaluating the downstream effects of the alterations in the levels of the proteins here considered may elucidate the biological pathways responsible for ALS and help discover novel biomarkers and therapeutic targets.

## Materials and methods

### Reagents

TDP-43 polyclonal antibody (10782-2-AP) was purchased from ProteinTech Group (Chicago, IL, USA). Matrin-3 (A300-591A) and FUS (A300-293A) polyclonal antibodies were from Bethyl Laboratories (Montgomery, TX, USA). C9orf72 (sc-138763), VCP (sc-20799), SOD1 (sc-11407), PARP-1 (sc-7150) polyclonal antibodies and GAPDH (sc-47724), profilin-1 (sc-137235) monoclonal antibodies, as well as ionomycin calcium salt (sc-3592), thapsigargin (sc-24017) and calpeptin (sc-202516) were obtained from Santa Cruz Biotechnology (Heidelberg, Germany). Anti-mouse IgG (7076P2) and anti-rabbit IgG (7074P2) antibodies conjugated to horseradish peroxidase (HRP) were from Cell Signaling Technology (Leiden, The Netherlands). The active human recombinant caspase set IV (K233-10- 25) was purchased from BioVision (Milpitas, CA, USA). Active calpain-1 (208712) and calpain-2 (208718) were from Calbiochem (La Jolla, CA, USA). MINI-PROTEAN TGX 4–15% precast gel (4561086SP5), TBT RTA Transfer Kit PVDF mini (1704272), Protein molecular markers (SM0671) and the RC DC Protein Assay Kit (500-0119) were purchased from Bio-Rad Laboratories (Milan, Italy). The WesternBright Enhanced chemiluminescent substrate (ECL) for HRP (K-12045-D50) was purchased from Advansta (Menlo Park, CA, USA). Nitrocellulose membranes (RPN303D) were purchased from Amersham (Milan, Italy). Lymphoprep^®^ (1114545) was purchased from Axis-Shield (Oslo, Norway). p62 (P0067) and LC3B (L7543) polyclonal antibodies, chloroquine (C6628-25G) as well as high grade versions of all other chemicals and cell media used in this study were purchased from Sigma-Aldrich (Milan, Italy).

### Cell culture

Human neuroblastoma SK-N-BE(2) adhesion cell lines (obtained from Biological Resource Center ICLC Cell bank, Core facility IRCCS Ospedale Policlinico San Martino, Genova) were cultured in RPMI 1640 supplemented with 10% foetal bovine serum (FBS), sodium pyruvate (1 ×) and an antibiotic cocktail (1 ×) at 37 °C and 5% CO_2_. Human carcinoma of the uterine cervix (HeLa, a kind gift of Prof. Marco Piccinini, University of Turin, Italy) cell lines were cultured in High glucose Dulbecco's Modified Eagle Medium (DMEM) supplemented with 10% FBS and an antibiotic cocktail (1 ×). PBMC from a healthy 35-year old donor (who signed a written informed consent) were collected in EDTA-coated tubes as previously described^66^ and then incubated in RPMI 1640 supplemented with 10% FBS and an antibiotic cocktail (1 ×). These three cell types were incubated in multiwell plates at 37 °C and 5% CO_2_.

### Treatment of SK-N-BE(2) lysate with calpains

SK-N-BE(2) cells (1 × 10^6^ cells for each sample) were lysed by repeated passage through a 26-G syringe in a reaction solution composed of 50 mM Tris–HCl pH 7.5, 30 mM NaCl, 5 mM DTT and 1 mM calcium chloride and then separately incubated with 2 U of active human calpain-1 or -2 for 10 min or 180 min at 37 °C. Furthermore, two samples were treated for 180 min with both calpain-1 or -2 and 20 mM calpeptin, a calpain inhibitor. The cleavage reaction was stopped by adding a buffer constituted by 50 mM Tris pH 6.8, 5% (w/v) SDS, 8 M deionized urea, and 2% (v/v) 2-mercaptoethanol and 10 mM EDTA. Samples were then frozen at − 80 °C.

### Treatment of SK-N-BE(2) lysate with caspases

SK-N-BE(2) cells (1 × 10^6^ cells for each sample) were lysed by repeated passage through a 26-G syringe in the reaction solution recommended by the manufacturer (50 mM HEPES pH 7.2, 50 mM NaCl, 0.1% CHAPS, 10 mM EDTA, 5% glycerol and 10 mM DTT) and then separately incubated with 2 U of active human caspase-3, -6, -7 or -8 for 180 min at 37 °C. The cleavage reaction was terminated by adding a buffer constituted by 50 mM Tris pH 6.8, 5% (w/v) SDS, 8 M deionized urea, and 2% (v/v) 2-mercaptoethanol. Samples were then frozen at − 80 °C.

### Cell treatments

SK-N-BE(2), HeLa cells and PBMC (1 × 10^5^ cells for each sample) were treated with ionomycin (1 µM or 5 µM) or thapsigargin (1 µM or 5 µM) for 24 h. Time-dependent experiments were carried out by incubating SK-N-BE(2) with 1 µM ionomycin or 1 µM thapsigargin for 2 h, 8 h and 24 h. Two-hour treatments of SK-N-BE(2) with 1 µM ionomycin or 1 µM thapsigargin were also performed following 2 h preincubation with 100 µM chloroquine.

### Western immunoblot analysis

Samples were subjected to SDS-PAGE using 4–15% precast gels as previously described^66^. Resolved proteins were then electro-transferred onto nitrocellulose membrane by using the Trans-Blot Turbo Blotting System (Bio-Rad) with the transfer buffer included in the TBT RTA Transfer Kit nitro mini supplemented with 20% (v/v) ethanol. Membranes were blocked with 2% bovine serum albumin (BSA) in a TBST buffer consisting of 0.02 M Tris–HCl pH 7.6, 0.14 M NaCl, and 0.02% (v/v) Tween 20. Membranes were then exposed to different antibodies in in TBST buffer with 5% BSA. Next, membranes were washed with TBST buffer, incubated with 15 ng/ml of appropriate HRP-conjugated secondary antibodies at 4 °C, washed again and then exposed to the enhanced chemiluminescence HRP substrate. The immunostained bands were visualized using a C-DiGit® Blot Scanner gel imaging system and Image Studio™ software ver 5.0 (LI-COR, Bad Homburg, Germany). When longer exposures were required, bands were detected using Amersham Hyperfilm ECL (GE Healthcare, Little Chalfont, UK).

### Statistical analysis

Statistical analyses were performed using SPSS software ver. 17.0 (IBM, Armonk, NY, USA). Data were analysed using the Student’s t-test. Significant differences were set at p < 0.05.

### Consent to participate and ethics approval

The two subjects whose PBMC were analysed in this study signed a written informed consent before blood drawn as a part of a study supported by the European Commission’s Health Seventh Framework Programme (FP7/2007–2013 under grant agreement 278611) and approved by the Ethics Committee of the Azienda Ospedaliero-Universitaria Città della Salute e della Scienza di Torino (protocol number 0025128 April 6, 2012) in accordance with the ethical standards laid down in the 1964 Declaration of Helsinki and its later amendments.

### Consent for publication

All authors have read and approved the submitted version of the manuscript.

## Supplementary Information

Supplementary Figures.

## Data Availability

All data generated or analysed during this study are included in this published article.
