# Supplementary material for: Effects of intracellular calcium accumulation on proteins encoded by the major genes underlying amyotrophic lateral sclerosis
Source: Sci Rep. 2022 Jan 10;12:395. doi: 10.1038/s41598-021-04267-8 (PMC8748718; doi:10.1038/s41598-021-04267-8)

Effects of intracellular calcium accumulation on proteins encoded by the major genes underlying amyotrophic lateral sclerosis

Giovanni De Marco[a,b,†,*], Annarosa Lomartire[a,†], Umberto Manera[a], Antonio Canosa[a,b], Maurizio Grassano[a], Federico Casale[a], Giuseppe Fuda[a], Paolina Salamone[a,b], Maria Teresa Rinaudo[c], Sebastiano Colombatto[c], Cristina Moglia[a,b], Adriano Chiò[a,b,d,e,†], Andrea Calvo[a,b,d,†]

[†]These authors equally contribute to this work

[a]ALS Centre, “Rita Levi Montalcini” Department of Neuroscience, University of Turin, Via Cherasco 15, 10126 Turin, Italy.
[b]Azienda Ospedaliero-Universitaria Città della Salute e della Scienza di Torino, Neurology Unit 1, Via Cherasco 15, 10126 Turin, Italy.
[c] Department of Oncology, University of Turin, via Michelangelo 27/b, 10126 Turin, Italy
[d]Neuroscience Institute of Turin (NIT), Via Verdi, 8, 10124 Turin, Italy.
[e]Institute of Cognitive Sciences and Technologies, C.N.R., Via S. Martino della Battaglia, 44, 00185 Rome, Italy.

*Corresponding author:
Giovanni De Marco
University of Turin
“Rita Levi Montalcini” Department of Neuroscience
Via Cherasco, 15
10126 Turin
Italy
+390116335439
giovanni.demarco@unito.it
ORCID ID 0000-0002-3966-8695

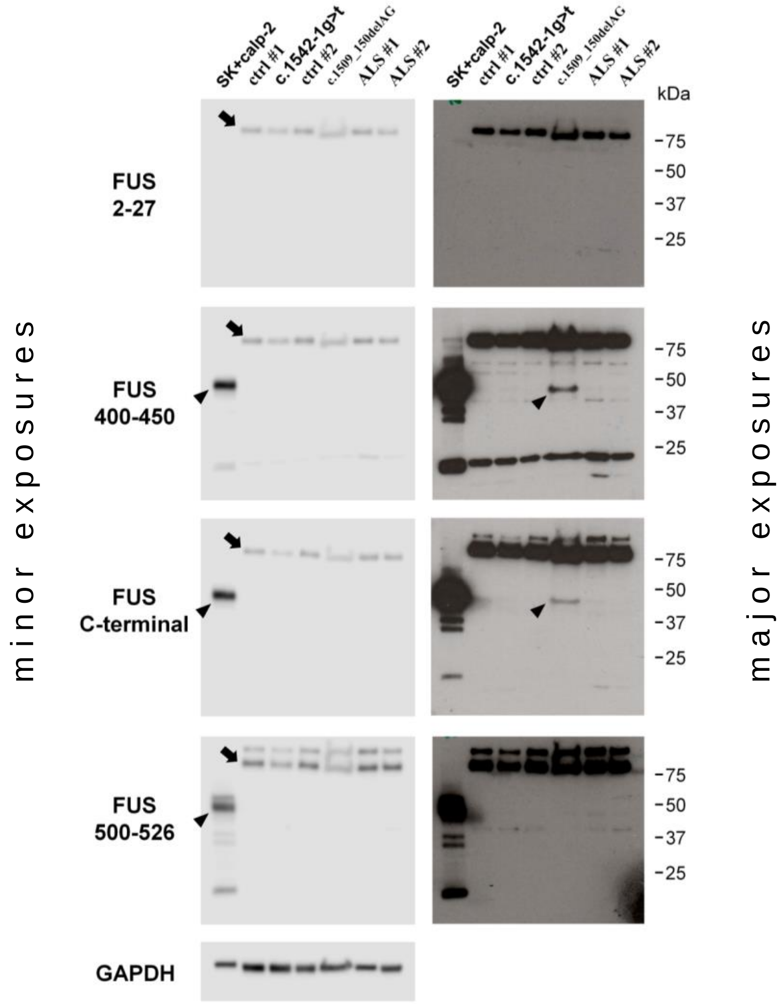

Figure S1 Cleavage of FUS in a patient carrying a mutation in the coding gene. FUS levels were evaluated using Western blot analysis in a lysate of SK-N-BE(2) treated with calpain-2, in PBMC from two neurologically-unaffected subject (ctrl #1 and #2), from two ALS patients not carrying *FUS* mutation (ALS #1 and #2), from an ALS patient with c.1542-1 g>t *FUS* mutation and from an ALS patient with c.1509_1510delAG *FUS* mutation. The antibodies used recognised four different epitopes: 2-27, 400-450, C-terminal and 500-526 of FUS protein. GAPDH was used as a measure of equal protein loading.
Arrow: full length FUS; arrowhead 45 kDa band.
The 45-kDa band obtained when SK-NBE(2) lysate was treated with calpain-2 was recognised by the antibodies 400-450, C-terminal and 500-526, but not by the antibody 2-27. This proteolytic product is thus a fragment lacking the N-terminal portion. A 45-kDa band was also clearly detected in PBMC of the ALS patient carrying the c.1509_1510delAG FUS mutation (described in Lanteri et al. 2021, PMID: 33637330). This band was here too recognised by the antibodies 400-450, 500-526 and not by 2-27, but also 500-526 was not able to detect it. The antibody 500-526 can not recognise the protein coded by the allele c.1509_1510delAG (see Lanteri et al. 2021). A possible explanation for this band is that it is a product derived from proteolysis by calpain (i.e. the same revealed in SK-N-BE(2) lysate), but deriving only from the product of the mutated allele. If this were the case, the protein codified by *FUS* carrying the c.1509_1510delAG mutation should be more susceptible to calpain activity than wild type protein.

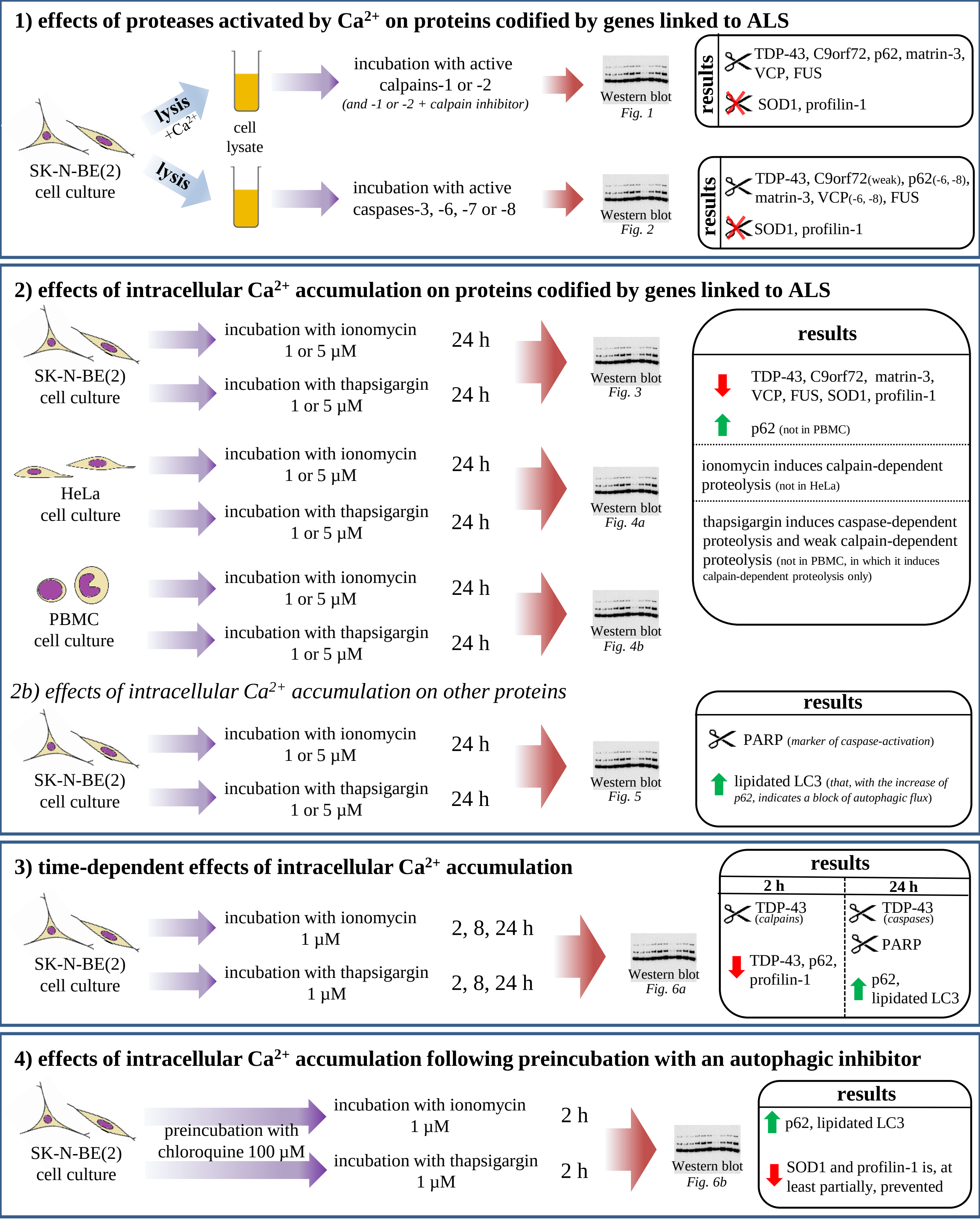

Figure S2 A summary of the experiments performed and the main results obtained.

Full-length Western immunoblot (1)

Fig. 1

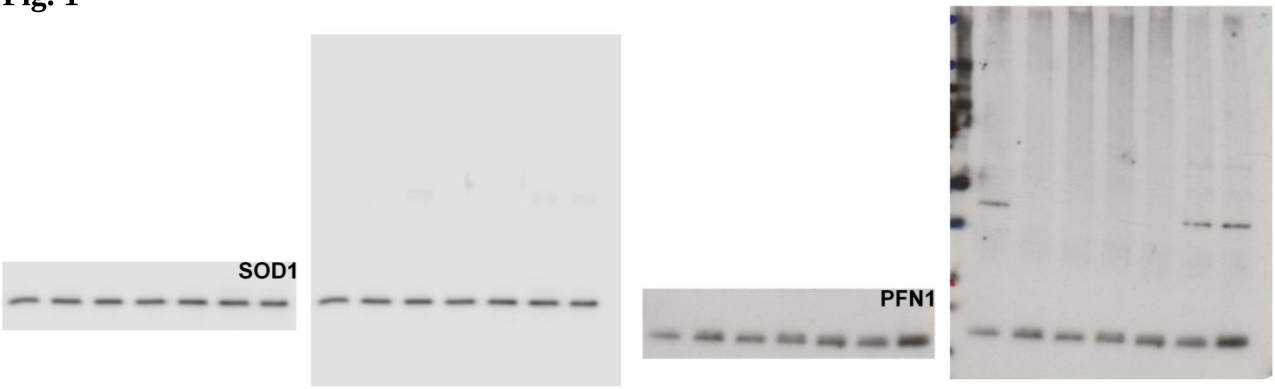

Fig. 2

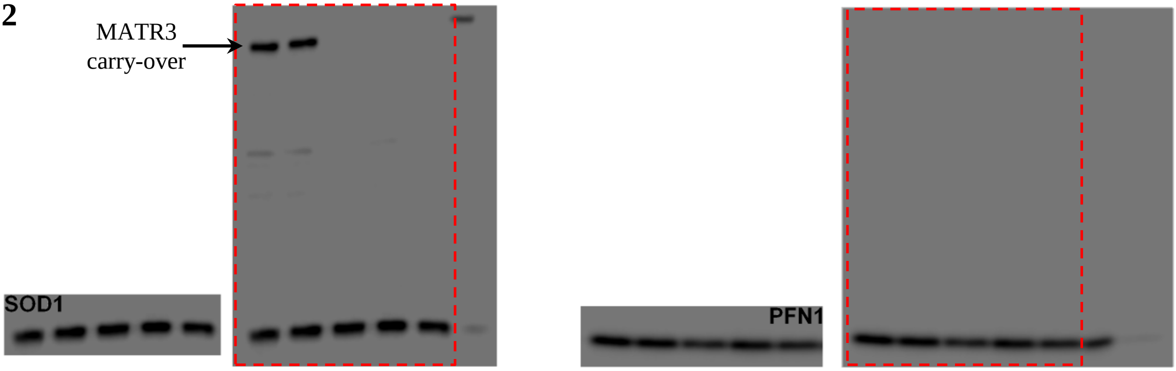

Fig. 3

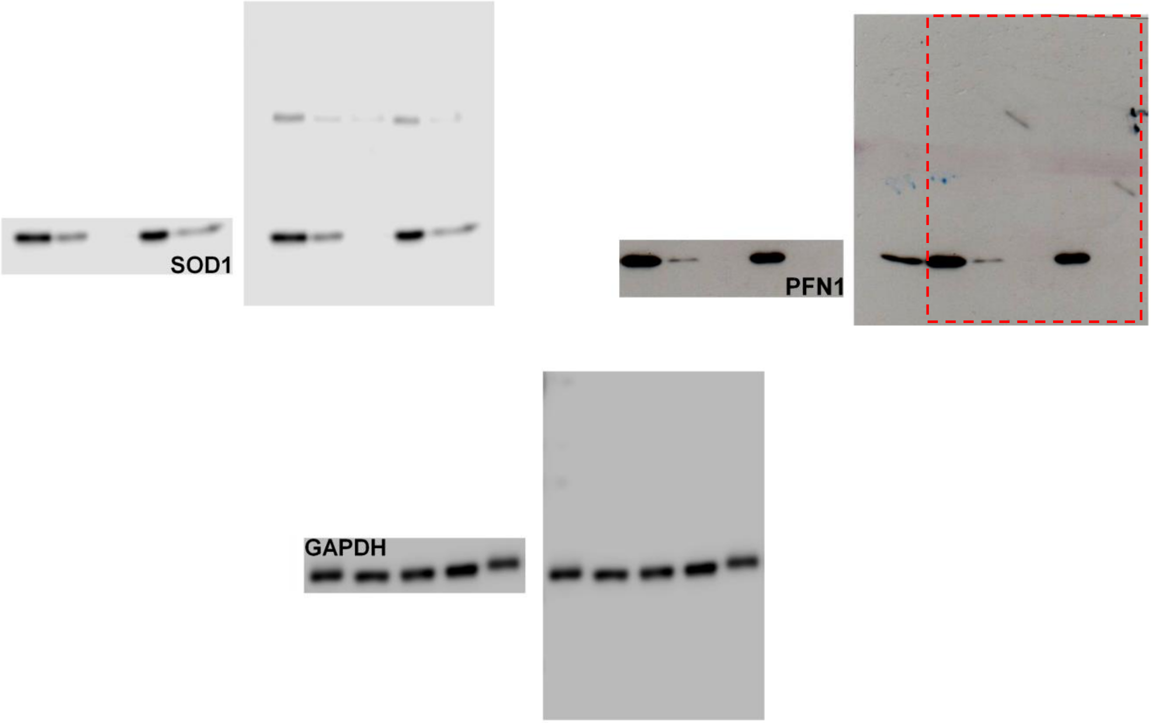

Full-length Western immunoblot (2)

Fig. 4a

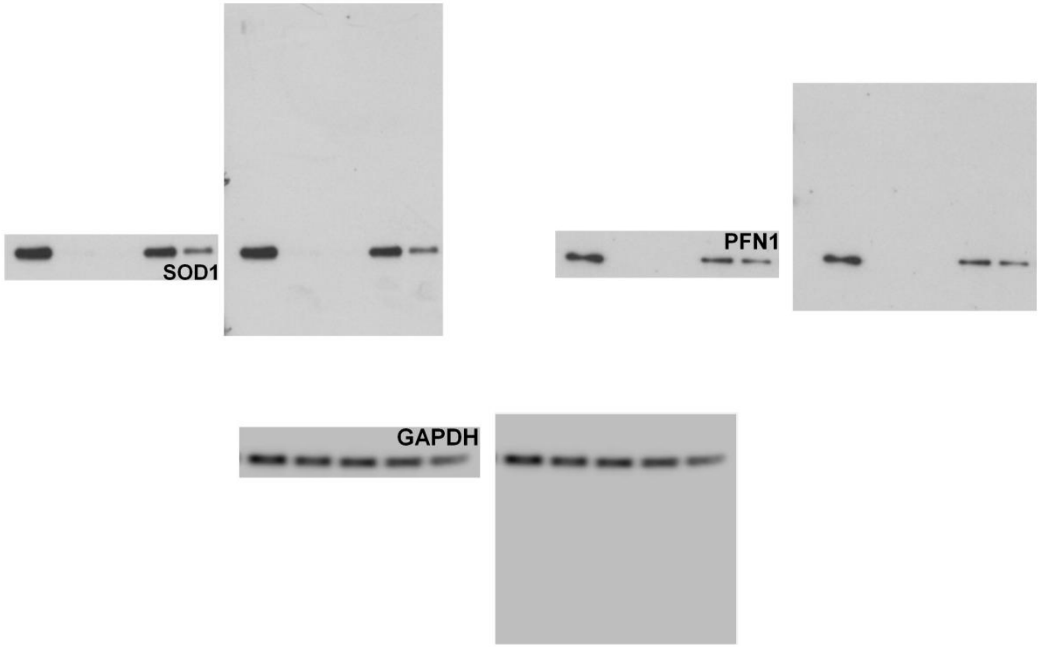

Fig. 4b

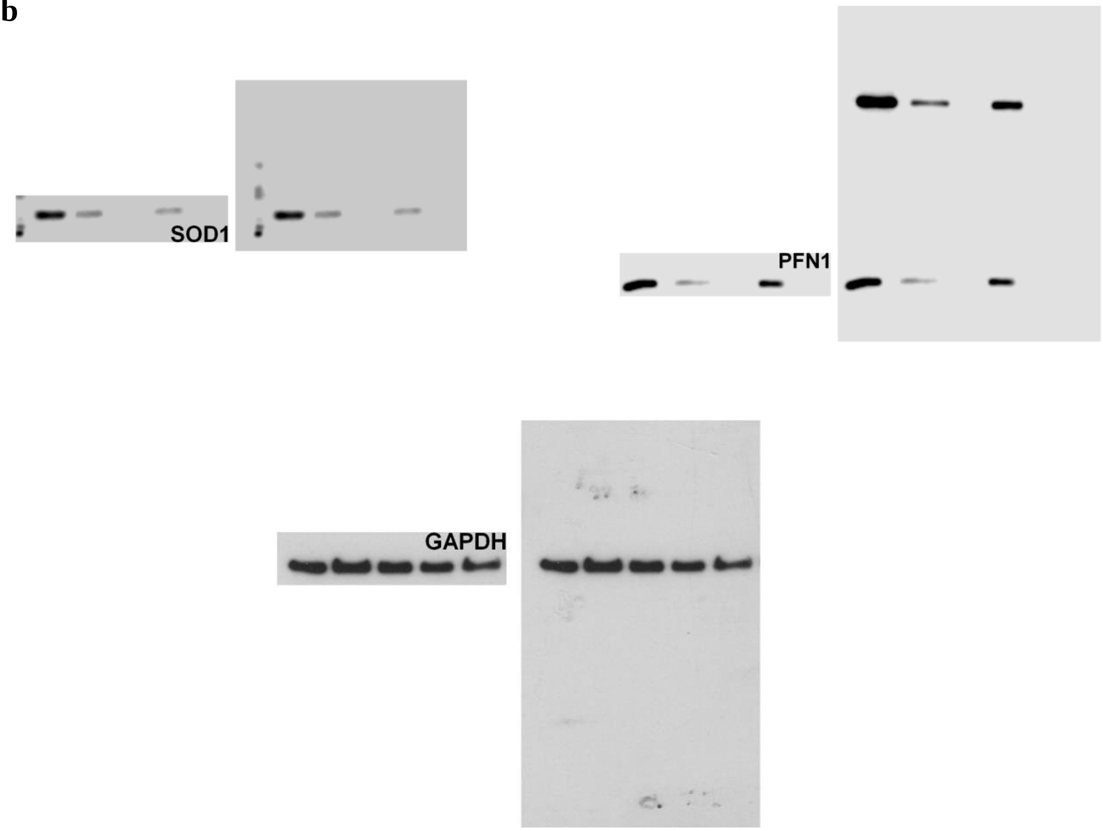

Full-length Western immunoblot (3)

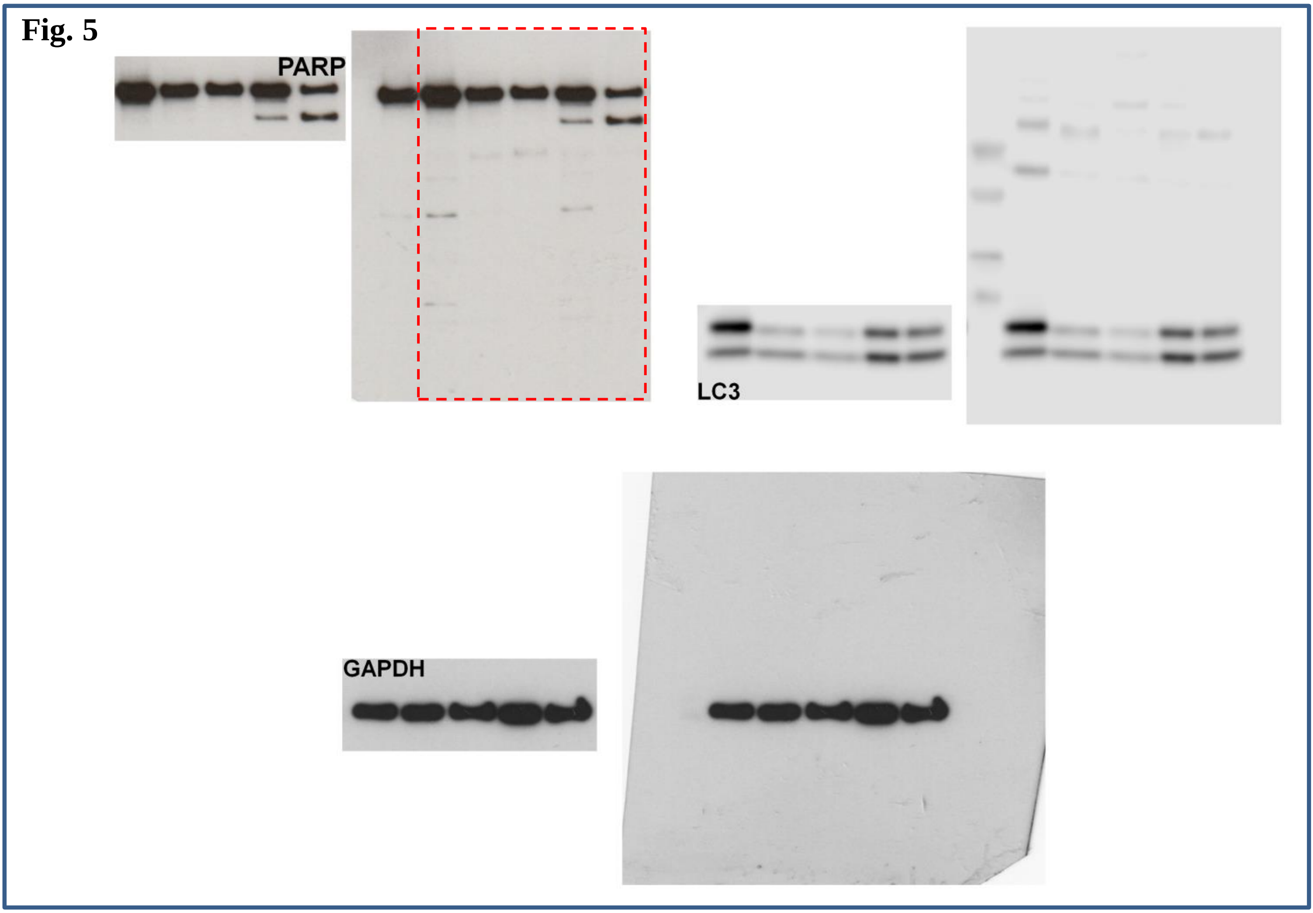

Full-length Western immunoblot (4)

Fig. 6a

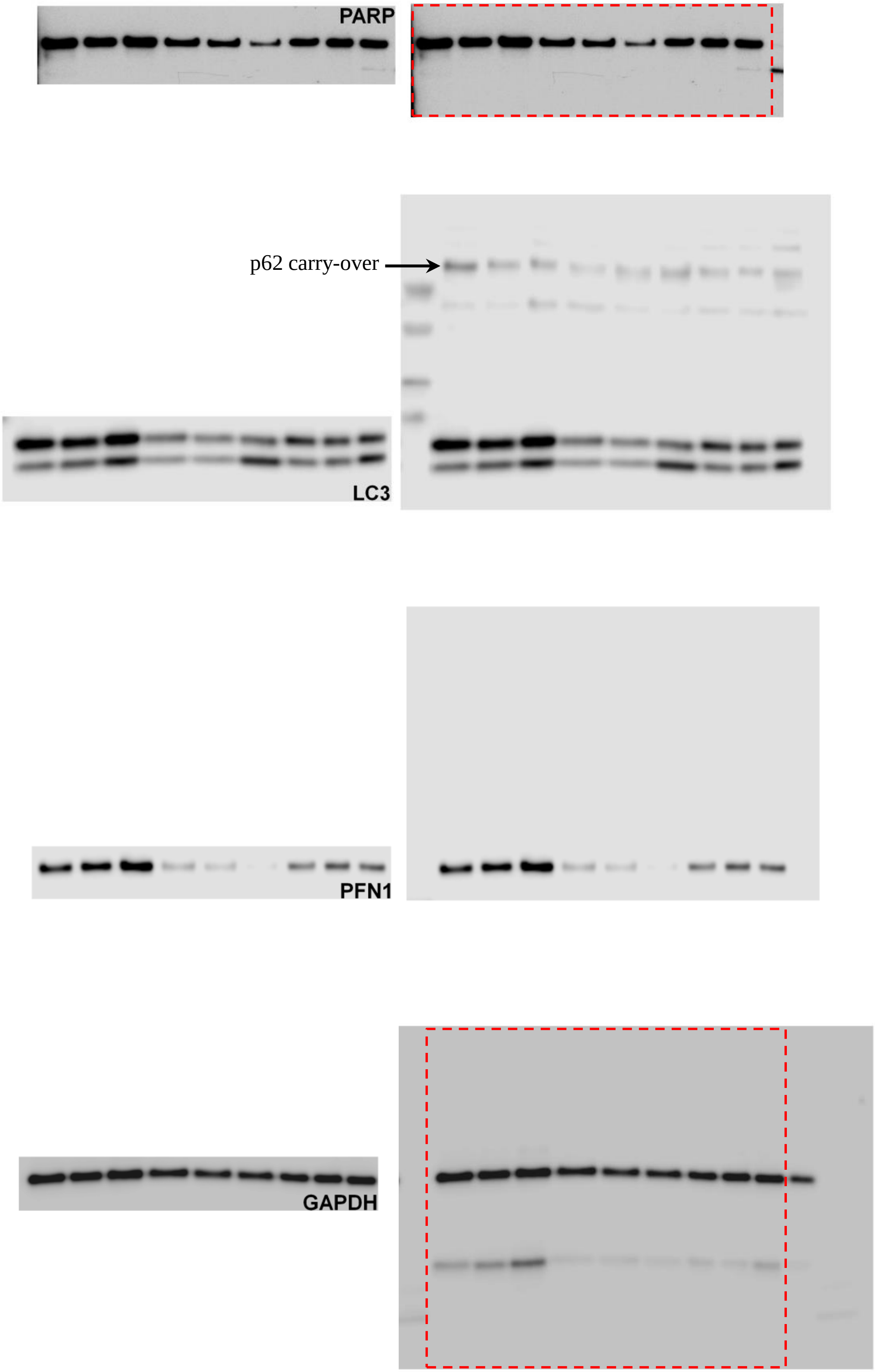

Full-length Western immunoblot (5)

Fig. 6b

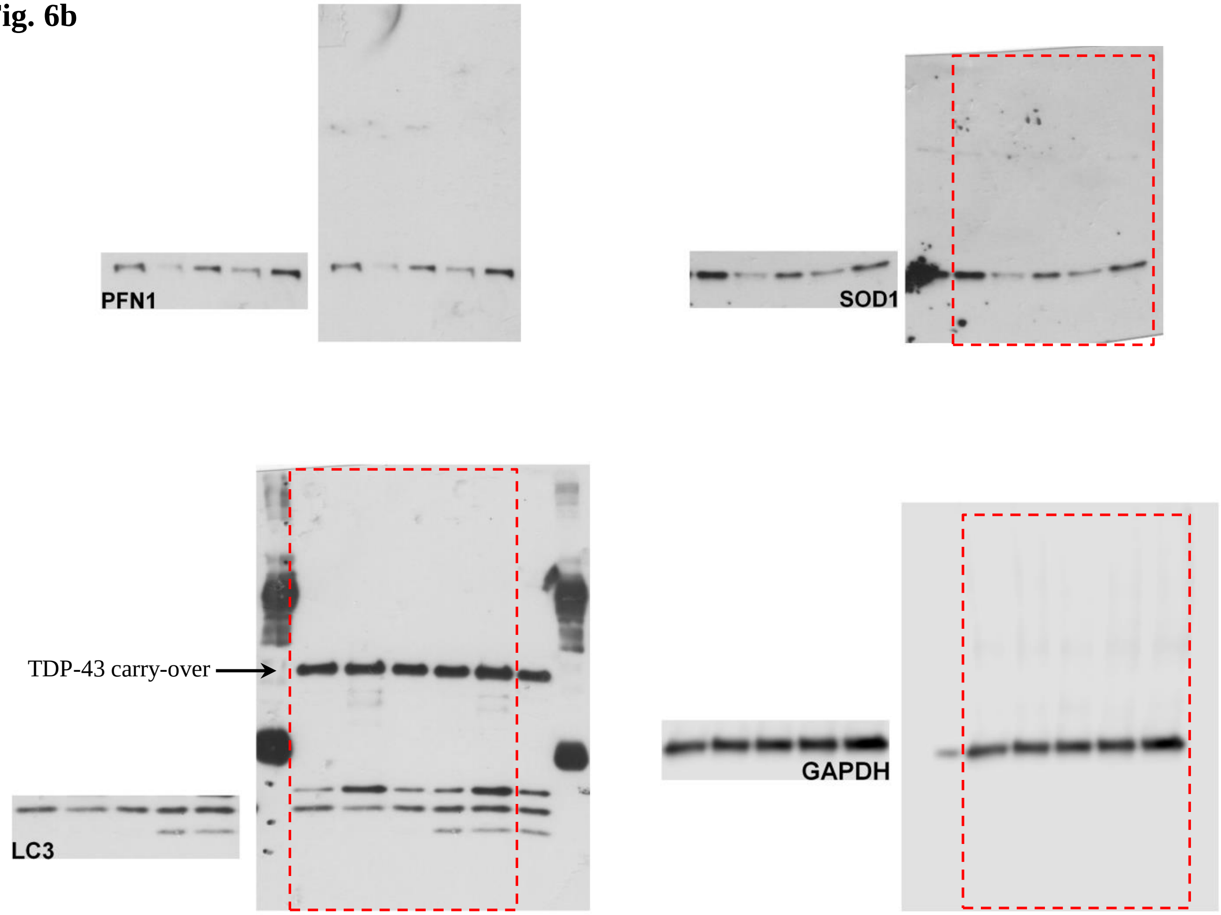

Fig. S1

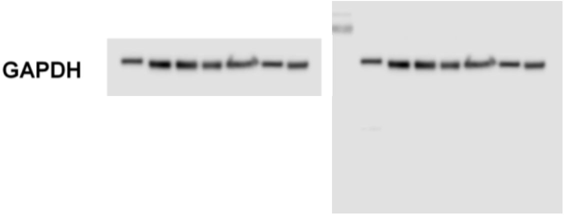

Supplement: Supplementary file 1 — Supplementary Figures. [file 41598_2021_4267_MOESM1_ESM.pdf]
